# Light Intensity—A Key Factor Affecting Flavonoid Content and Expression of Key Enzyme Genes of Flavonoid Synthesis in Tartary Buckwheat

**DOI:** 10.3390/plants11162165

**Published:** 2022-08-21

**Authors:** Di Wang, Tao Yang, Yangqian Li, Fang Deng, Shuai Dong, Wei Li, Yueyue He, Jinming Zhang, Liang Zou

**Affiliations:** 1 School of Pharmacy, Chengdu University of Traditional Chinese Medicine, Chengdu 611137, China; 2 School of Intelligent Medicine, Chengdu University of Traditional Chinese Medicine, Chengdu 611137, China; 3 Asset and Laboratory Management Department, Chengdu University of Traditional Chinese Medicine, Chengdu 611137, China; 4 School of Basic Medicine, Chengdu University, Chengdu 610106, China; 5 Key Laboratory of Coarse Cereal Processing, Ministry of Agriculture and Rural Affairs, Chengdu University, Chengdu 610106, China

**Keywords:** tartary buckwheat, flavonoids content, light intensity, key enzyme gene, seed coat color

## Abstract

Tartary buckwheat, a polygonaceae family plant, is rich in abundant flavonoids, high-quality protein, and well-balanced essential amino acids. This study aimed to investigate the effects of climatic variables on the quality of Tartary buckwheat. In this study, six distinct types of Tartary buckwheat collected from the Sichuan Basin, Western Sichuan Plateau, and Yunnan-Guizhou Plateau in southwest China were chosen to investigate the impact of climatic conditions from the grain-filling stage to the harvest stage on the concentration of flavonoids and expression of key enzyme genes involved the synthesis of flavonoids. Meteorological data of three producing areas were collected from the China Meteorological Network, mainly including maximum temperature (Tmax), minimum temperature (Tmin), diurnal temperature difference (Tdif), and light intensity. Then, the contents of rutin, kaempferol-3-O-rutin glycoside, quercetin, and kaempferol in 30 batches of Tartary buckwheat from 6 varieties including Chuanqiao No. 1, Chuanqiao No. 2, Xiqiao No. 1, Xiqiao No. 2, Miqiao No. 1 and Di ku were determined by ultra performance liquid chromatography-mass spectrometry (UPLC-MS/MS). Furthermore, the expression levels of phenylalanine ammonia lyase (PAL), 4-coumaric acid coenzyme A ligase (4CL), and anthocyanin synthase (ANS) in six kinds of Tartary buckwheat were detected by real-time polymerase chain reaction (PCR). The seed photos were processed by ImageJ processing software. The partial least squares method was used to analyze the correlation. As a result, light intensity can promote the accumulation of flavonoids and the expression of key enzyme genes. Miqiao No. 1, which grows in Liangshan Prefecture, Sichuan Province, has the highest light intensity and is the dominant variety with flavonoid content. More importantly, the expression levels of PAL and 4CL in the secondary metabolic pathway of flavonoids were positively correlated with the content of Tartary buckwheat flavonoids. Interestingly, the expression level of ANS was negatively correlated with the content of PAL, 4CL, and flavonoids. In addition, ANS is a key gene affecting the seed coat color of Tartary buckwheat. The higher the expression of ANS, the darker the seed coat color. These findings provide a theoretical basis and reference for the breeding of fine buckwheat varieties.

## 1. Introduction

**Figure 1:**
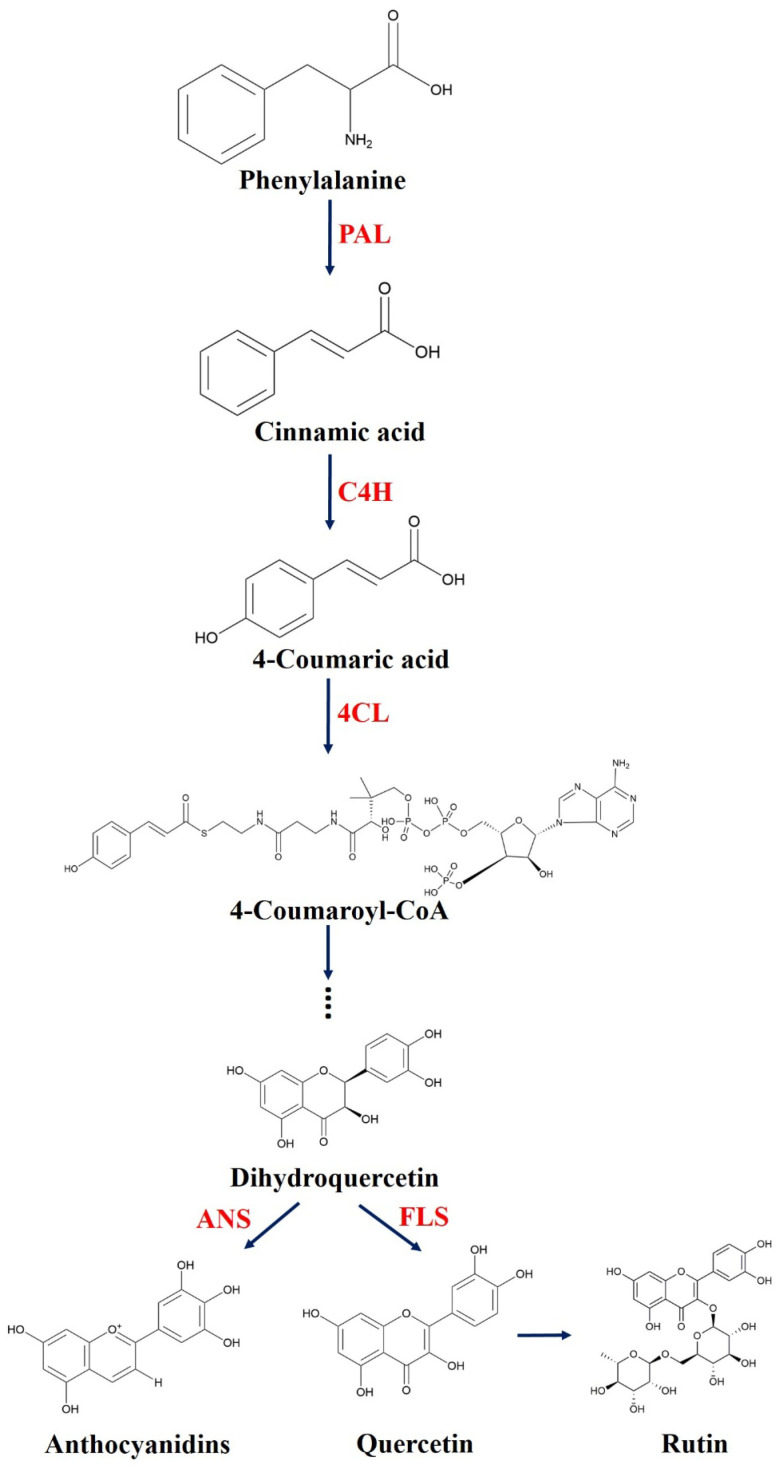
Schematic diagram of flavonoid anabolism in plants [13]. Note: PAL: Phenylalanine ammonia lyase; C4H: Cinnamate -4-hydroxylase; 4CL: 4-coumaric acid coenzyme A ligase; ANS: Anthocyanin synthase; FLS: Plant flavonol synthase.

Tartary buckwheat (*Fagopyrum tataricum* (L.) *Gaertn*), as a gluten-free and health preservation crop, has been traditionally used as a staple food for centuries in southwest China, Tibetan border areas, and also in other high-altitude areas [1,2,3]. Tartary buckwheat is particularly rich in rutin, quercetin, kaempferol, and many other bioactive substances which can relieve a variety of human sub-health symptoms [4]. This can be illustrated briefly by protecting the liver from damage, oxidative stress, and vascular disease [4,5]. Additionally, flavonoids are unique secondary metabolites of Tartary buckwheat, and their biosynthesis pathway is mainly the phenyl propane pathway (Figure 1) [6]. PAL, the first key enzyme in the phenyl propane pathway, is the catalyzed non-oxidative deamination of L-phenylalanine to trans-cinnamic acid. Meanwhile, 4CL, as a key rate-limiting enzyme in the phenyl propane pathway, can catalyze coumaric acid and cinnamic acid to form coumaryl-CoA and cinnamyl-CoA, respectively. Moreover, dihydroflavone, which is the main precursor of other flavonoids, is converted to rutin and quercetin by FLS and to anthocyanins by ANS [7,8,9,10,11,12]. 

It is well known that the geographical environment has a great influence on the quality of Tartary buckwheat. Especially in southwest China, because of its complicated geographical environment and diverse climate types, it has been recognized as the origin and diversity center of buckwheat in the world [3]. It has been manifested that light intensity, low-temperature stress, and different altitudes can affect plant biochemistry, gene expression, and metabolic regulation [14,15]. Song et al. [15] explained the different cold adaptation strategies of Tartary buckwheat at different altitudes at the molecular level. Zhu et al. [16] irradiated Tartary buckwheat seedlings with ultraviolet light and found that the contents of rutin, catechin, and epatechin in Tartary buckwheat were significantly increased after the UV exposure. However, the knowledge about the correlation between flavonoid content, seed coat color, key enzyme gene expression, and the growth environment for Tartary buckwheat was still limited. Therefore, in this study, six Tartary buckwheat varieties from three different ecological environments in southwest China were taken as the research objects. UPLC-MS/MS, real-time PCR, and partial least squares regression (PLS) methods were used to clarify the influence of climatic factors on the content of Tartary buckwheat flavonoids and the expression of key enzyme genes in their synthesis pathways. The findings from the present study can provide a theoretical basis for the planting and breeding of Tartary buckwheat with high quality.

## 2. Results and Discussion

### 2.1. Analysis of Meteorological Data in Different Producing Areas

**Figure 2:**
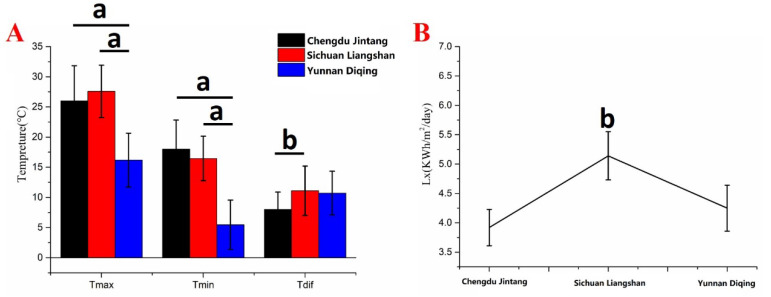
The average maximum temperature (Tmax), minimum temperature (Tmin), and diurnal temperature difference (Tdif) of each area (**A**) and the average light intensity in each area (**B**). These values were calculated from 1 March to 31 August 2021. Note: ^a^ *p* < 0.01, with significant difference, ^b^ *p* < 0.05, with extremely significant difference.

The results demonstrated that the average Tmax and Tmin of Jintang in Chengdu and Liangshan in Sichuan were significantly higher than those of Diqing in Yunnan (*p* < 0.01) (Figure 2A). The average Tdif in Liangshan was significantly greater than that in Jintang, Chengdu (*p* < 0.05). In addition, the average light intensity in Liangshan Prefecture of the western Sichuan Plateau is the strongest, which is 1.31 and 1.21 times that of Jintang and Diqing, respectively, and there are significant differences (*p* < 0.05) (Figure 2B). 

### 2.2. Analysis of Four Flavonoid Components in Different Varieties of Tartary Buckwheat

#### 2.2.1. Total Ion Flow Diagram and Secondary Mass Spectrometry of Flavonoids in Different Varieties of Tartary Buckwheat

**Figure 3:**
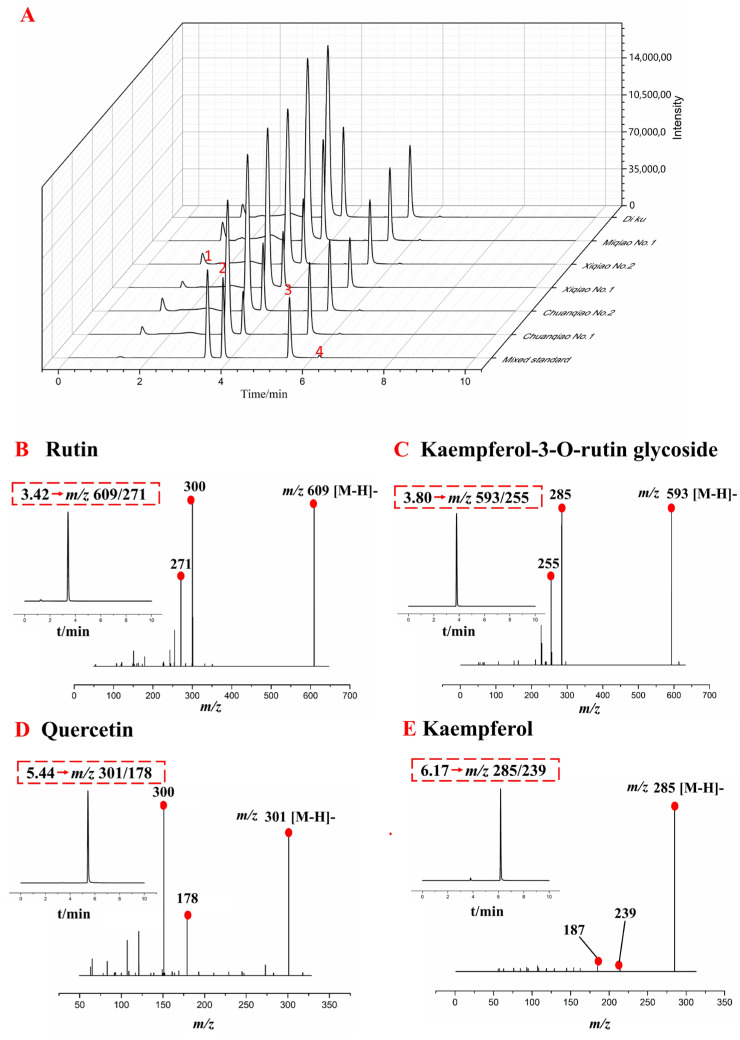
(**A**) Total ion flow diagram of different varieties of Tartary buckwheat, (**B**–**E**) primary and secondary mass spectrometry information of four flavonoids. Note: 1-Rutin, 2-Kaempferol-3-O-rutin glycoside, 3-Quercetin, 4-Kaempferol.

Four flavonoids were detected by multiple reaction monitoring (MRM) mode in the mixed reference and six different varieties of Tartary buckwheat. The retention time of rutin, kaempferol-3-O-rutin, quercetin, and kaempferol in the total ion flow diagram were 3.42, 3.80, 5.44, and 6.17 min, respectively. The MRM mode total ion flow diagram and extraction ion flow secondary mass spectrometry information of the four flavonoids were shown in Figure 3. The results showed that the total ion flow diagram is basically the same (Figure 3A). Each sample was repeated three times, showing good repeatability. 

#### 2.2.2. Cluster Analysis of Content Difference in Their Own Metabolites

**Figure 4:**
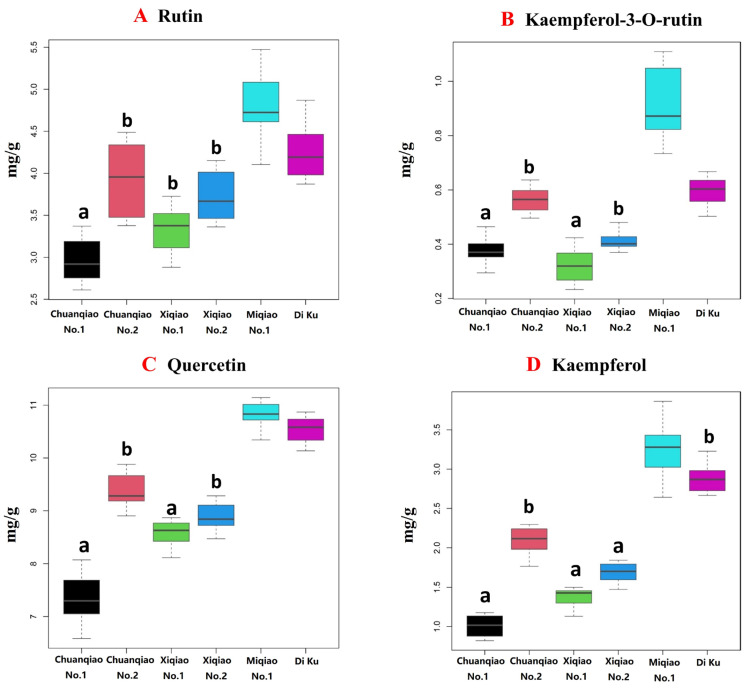
Box plot of contents of four flavonoids in different varieties of Tartary buckwheat. (**A**–**D**) contents of rutin, kaempferol-3-O-rutin glycoside, quercetin and kaempferol in six varieties of Tartary buckwheat. Note: Compare to Miqiao No.1: ^a^ *p* < 0.01, ^b^ *p* < 0.05.

The contents of four flavonoids in Tartary buckwheat were significantly different in different areas (Figure 4A–D). The contents of these components can be divided into two main subgroups, among which Miqiao No. 1, Chuanqiao No. 2, and Di ku are one cluster, and Chuanqiao No. 1, Xiqiao No. 1, and Xiqiao No. 2 are another cluster. In the group represented by Miqiao 1, the total contents of four flavonoids were 54.07 ± 1.02 mg∙g^−1^, including 30.74 ± 0.78, 13.02 ± 0.45, 8.23 ± 0.19, and 2.08 ± 0.45 mg∙g^−1^ of rutin, quercetin, Kaempferol-3-O-rutin glycoside, and Kaempferol, respectively. However, the total contents of four flavonoids in the group represented by Chuanqiao1 were only 39.99 ± 1.46 mg∙g^−1^, including 24.78 ± 0.82, 10.04 ± 0.38, 4.06 ± 0.33, and 1.11 ± 0.05 mg∙g^−1^ of rutin, quercetin, Kaempferol-3-O-rutin glycoside, and Kaempferol, respectively (Figure 4). The contents of total flavonoids and components in Miqiao No. 1 were 1.24, 1.35, 1.30, 2.03, and 1.87 times that in Chuanqiao No. 1, respectively. These findings indicated that Miqiao No. 1 was the dominant variety of flavonoid content. 

The results also showed that the content of rutin in six kinds of Tartary buckwheat was the highest, which was consistent with previous reports [17]. However, the difference in flavonoid content among varieties was obvious, and the flavonoid content of Miqiao No. 1 growing in Liangshan Prefecture with high altitude and strong sunlight was the highest. Moreover, it has been reported that light-induced transcription factor FtMYB116 can promote the accumulation of rutin in Tartary buckwheat [18]. Therefore, we speculated that Miqiao No. 1 was the dominant variety of flavonoid content, which may be related to the meteorological environment.

### 2.3. Expression Analysis of Key Enzyme Genes in Different Varieties of Tartary Buckwheat

The synthesis of flavonoids in Tartary buckwheat is mainly a phenylpropanoid metabolic pathway, which is related to PAL, 4CL, and chalcone synthase (CHS), as well as anthocyanin synthase ANS [19]. PAL, the first key enzyme in the phenyl propane pathway, catalyzed the non-oxidative deamination of L-phenylalanine to trans-cinnamic acid. 4CL, as a key rate-limiting enzyme in the phenyl propane pathway, can catalyze coumaric acid and cinnamic acid to form coumaryl-CoA and cinnamyl-CoA, respectively. The high expression of PAL and 4CL may promote the accumulation of flavonoids. Moreover, dihydroflavone, which is the main precursor of other flavonoids, is converted to rutin and quercetin by FLS and to anthocyanins by ANS [13]. However, due to PAL, 4CL, and ANS sharing a common substrate “dihydroflavone”, the high expression of ANS can promote the generation of anthocyanidin and thus reduce the accumulation of flavonoids. 

**Figure 5:**
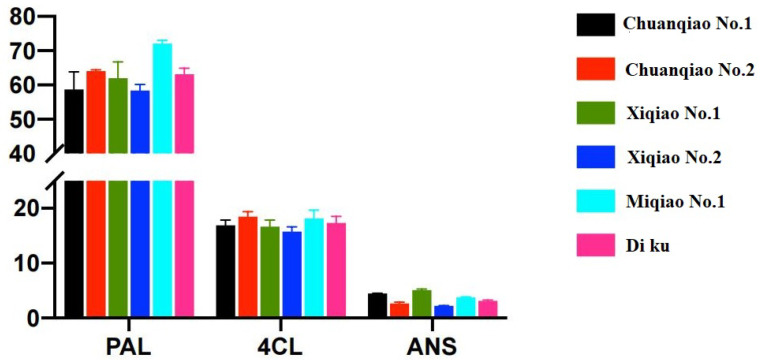
Expression analysis of key enzyme genes in different varieties of Tartary buckwheat. Note: There was no significant difference in the expression of three genes in different varieties of Tartary buckwheat (*p* > 0.05).

As shown in Figure 5, the expression levels of PAL and 4CL in six varieties were accordant, and those of Miqiao No. 1 were all the highest, but there was no significant difference (*p* > 0.05). However, the expression of the ANS gene was not in accordance with that of PAL and 4CL. A possible explanation may be a common substrate “dihydroflavone” they shared in the flavonoid synthesis pathway [13]. For this purpose, the relationship between gene expression and flavonoid content was further analyzed in this study. 

### 2.4. Correlation Analysis of Enzyme Gene Expression and Content in Tartary Buckwheat

**Figure 6:**
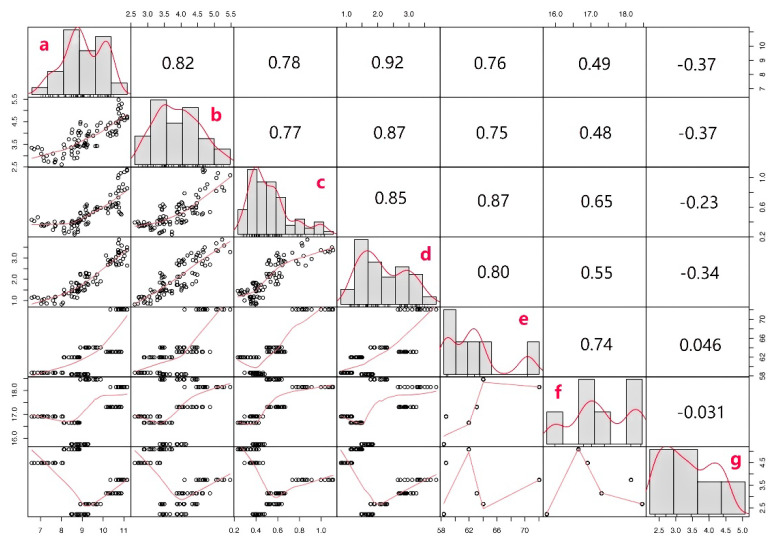
Relationship between flavonoid key enzyme gene expression and content in Tartary buckwheat. Note: a: Rutin, b: Quercetin, c: Kaempferol, d: Kaempferol-3-O-rutin glycoside, e: PAL, f: 4CL, g: ANS.

The correlation of four flavonoid components and the expression of three key enzyme genes was analyzed in Figure 6. A strong correlation is apparent between the four flavonoids content and the expression of PAL and 4CL (Figure 6, lines 1 to 6), indicating the gene expression of PAL and 4CL can promote the accumulation of flavonoids. The expression of ANS was negatively correlated with the expression of PAL, 4CL, and the content of flavonoids. This might be because PAL, 4CL, and ANS share a common substrate “dihydroflavone”. Dihydroflavone, which is the main precursor for flavonoids, is converted to rutin and quercetin by PAL and 4CL, and to anthocyanins by ANS [13]. Furthermore, since the expression levels of PAL and 4CL are highly consistent with the contents of the four flavonoids, researchers only need to select one of PAL and 4CL. 

Lines 1 to 4 indicated the content of rutin, quercetin, kaempferol, and kaempfer-3-O-rutin glycoside, respectively. Lines 5 to 7 indicated the gene expression levels of PAL, 4CL, and ANS, respectively. In addition, the main diagonal represented the correlation of any two indexes, and the upper triangular matrix represented the correlation value.

### 2.5. Effects of Meteorological Factors on the Expression of the Key Enzyme Gene of Flavonoid

The PLS model finally selected the model with interactive terms. As shown in Table 1, it could explain the 63.29% variance of PAL, 4CL, and ANS gene expression differences. X represents four meteorological factors including Tmax, Tmin, Tdif, and light intensity, and Y represents the expression levels of three genes. This finding also indicated that light intensity was the key factor affecting the expression of flavonoid-related enzyme genes in Tartary buckwheat (light intensity coefficient: 1.51). Cheng. et al. [20] also confirmed that light intensity significantly upregulated the relative expression levels of CHS, FLS, and PAL in Tartary buckwheat buds, of which the results were consistent with this study. It indicates that stress treatment by increasing light intensity may promote the expression of flavonoids related enzyme genes. Therefore, using strong light stimulation might be a promising way to breed new varieties of Tartary buckwheat.

The result of “2.4” showed that the flavonoid content was positively correlated with PAL and 4CL expression. In order to verify the conjecture that meteorological factors can directly affect flavonoid content, the PLS model was also used in this study for further verification. 

The PLS model with no interaction term and no quadratic term was finally selected. As shown in Table 2, it could explain the variance of 60.45% of flavonoid content from different producing areas. X represents four meteorological factors including Tmax, Tmin, Tdif, and light intensity, and Y represents the contents of four flavonoids. PLS regression showed that light intensity was the key factor affecting the flavonoid content of Tartary buckwheat (light intensity coefficient: 1.17). Liangshan Prefecture in Sichuan province, where Miqiao No. 1 is located, has the highest average light intensity, reaching 5.14 ± 0.66 KWh/m2/day, and with the highest total flavonoid contents. Zhang et al. [21] analyzed and compared the content of flavonoids in Tartary buckwheat from Shaanxi, Yunnan, Guizhou, and Sichuan, and found that the content of flavonoids from Yunnan with high altitude and a strong light was significantly higher than that from other areas.

The results of this study further verified that light intensity can improve the expression of flavonoid-related enzyme genes, and thus promote the accumulation of flavonoids in Tartary buckwheat.

### 2.6. Effects of Flavonoid Key Enzyme Gene Expression on Seed Coat Color

**Figure 7:**
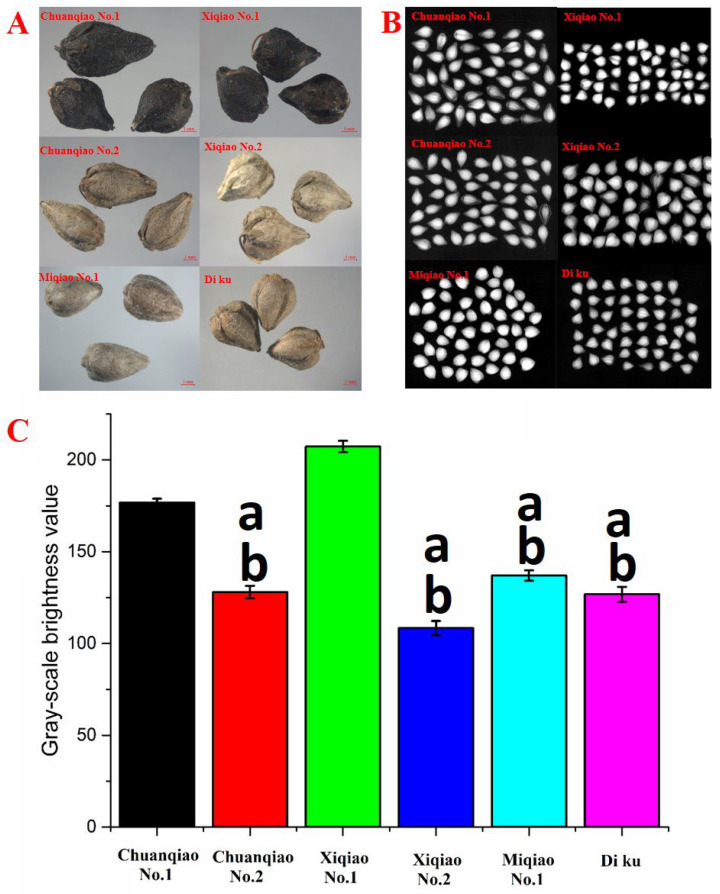
Seed morphological characteristics of different varieties of Tartary buckwheat. (**A**) Seed images under a microscopic imaging system, (**B**) seed X-ray images under an X-ray digital imaging system, and (**C**) grayscale brightness value. Note: Chuanqiao No. 1 VS. Chuanqiao No. 2, Xiqiao No. 2, Miqiao No. 1, and Di Ku, ^a^ *p* < 0.05; Xiqiao No. 1 VS. Chuanqiao No. 2, Xiqiao No. 2, Miqiao No. 1 and Di ku, ^b^ *p* < 0.05.

Six different varieties of Tartary buckwheat exist with obvious color differences (Figure 7A). Chuanqiao No. 1 and Xiqiao No. 1 are mainly black, while the other four buckwheat varieties are mainly grayish brown. There were significant differences in the brightness of grayscale among six varieties of Tartary buckwheat (Figure 7C). Zhang et al. [3]. mined genetic enzymes associated with Tartary buckwheat seed coat color by genome-wide association analysis (GWAS). Accordingly, we suspected that the seed coat color of Tartary buckwheat might be related to the expression of related gene enzymes. Surprisingly, this result indicated that the gray-scale brightness value of six varieties of Tartary buckwheat seed coat color was in line with ANS gene expression. The findings are more attractive for further research. 

The PLS model was also used to further analyze the relationship between flavonoid key enzyme gene expression and seed coat color. The PLS model with no interaction term and no quadratic term was finally selected. As can be seen from Table 3, it could explain the 89.41% variance of different Tartary buckwheat seed coat color. X represents the expression levels of PAL, 4CL, and ANS genes, and Y represents the color difference of different varieties of Tartary buckwheat. These results suggested that the ANS gene is the key factor affecting the coat color of Tartary buckwheat (ANS coefficient: 31.94), indicating that the higher the expression of ANS, the darker the coat color of Tartary buckwheat. Wei. et al. [22] established a targeted metabolomics method based on liquid mass spectrometry to analyze and identify the chemical components of flavonoids and anthraquinones in Tartary buckwheat seeds and discussed the relationship between the morphology and color differences of Tartary buckwheat grains and husks and their metabolites. The results showed that 12 flavonoids and 3 anthraquinones in Tartary buckwheat grains were related to seed color, and 11 flavonoids were related to morphology. In addition, 16 flavonoids and 2 anthraquinones in Tartary buckwheat husk were correlated with seed color, and two flavonoids were correlated with the morphology.

## 3. Materials and Methods

### 3.1. Materials and Reagents

Chuanqiao No. 1, Chuanqiao No. 2, Xiqiao No. 1, and Xiqiao No. 2 (*Fagopyrum tataricum* (L.) *Gaertn*) were all collected from Jintang, Sichuan province. Miqiao No. 1 and Di ku (*Fagopyrum tataricum* (L.) *Gaertn*) used in this study were obtained from Liangshan, Sichuan province and Diqing, Yunnan province, respectively. Five batches of Tartary buckwheat seeds with seed coats were collected from each producing area, and the sample information is shown in Table 4. Rutin, kaempferol-3-O-rutin glycoside, quercetin, and kaempferol were purchased from Chengdu Desite Biotech Co., Ltd. (Chengdu, China). All reference substances had a purity of ≥98%. Acetonitrile (HPLC grade) and formic acid (HPLC grade) were purchased from Fisher (Fisher Scientific, New York, NY, USA). Distilled water was purchased from Watsons Corporation (Hong Kong, China).

### 3.2. Meteorological Data Collection

Meteorological data were collected from China Meteorological Network. Since the Tartary buckwheat planted in southwest China generally concentrates from March to August from the filling stage to the harvest stage, meteorological data of three producing areas from March to August 2021 were collected, including Tmax, Tmin, Tdif, and light intensity.

- Tmax: daily maximum temperature (Statistics from 1 March to 30 August 2021 in China Meteorological Network)
- Tmin: daily minimum temperature (Statistics from 1 March to 30 August 2021 in China Meteorological Network)
- Tdif: diurnal temperature difference (the difference between Tmax and Tmin, statistics from 1 March to 30 August 2021)
- Light intensity: the average amount of solar radiation per square meter per day (Statistics from 1 March to 30 August 2021 in China Meteorological Network).

### 3.3. Extraction Procedure

An accurate amount of Chuanqiao No. 1, Chuanqiao No. 2, Xiqiao No. 1, Xiqiao No. 2, Miqiao No. 1, and Di ku (0.4 g) were placed into a conical flask, respectively, adding 10 mL of 75% ethanol, and extracted with an ultrasonic bath for 30 min [2] (JDUT-2 ultrasonic cleaning apparatus, power 500 W, frequency 40 kHz) at room temperature. The sample solution was filtered through a 0.22 μm filtering membrane and stored in a 4 °C refrigerator for later use.

### 3.4. Preparation of Mixed Authentic Standards Solution

Each reference substance was accurately weighed and dissolved in 10 mL of methanol to make a single reference reserve solution. In addition, a series of mixed reference solutions of different concentrations were obtained by gradual dilution. Then, the solution was filtered through a 0.22 μm of filtering membrane before injection.

### 3.5. Determination of Four Flavonoid Components in Different Varieties of Tartary Buckwheat by UPLC-MS/MS

#### 3.5.1. Chromatographic and Mass Spectrometry Conditions

The chromatographic separation was performed on a Thermo Scientific Accucore^TM^ C_18_ (100 mm × 3 mm, 2.6 μm) column using water—0.1% formic acid solution (eluent A) and acetonitrile (eluent B) as the mobile phase at a flow rate of 0.3 mL·min^−1^. The gradient program was set as follows: 0–10 min, 15–90% B. The column temperature was 30 °C, and the injection volume was 5 μL.

The MS data were acquired using electrospray ionization (ESI) under the negative ions scan modes; spray voltage, −3.5 kV; sheath gas, 35 arb; auxiliary gas, 15 arb; heater temperature, 350 °C; capillary temperature, 350 °C. Scanning mode: MRM. The compound optimization parameters are shown in Table 5. 

#### 3.5.2. Method Validation

The calibration curves for 4 compounds were calculated using their peak areas (y) and concentrations (x). The limit of detection (LOD) and limit of quantification (LOQ) of 4 compounds were evaluated at signal-to-noise ratios (S/N) of 3 and 10. All calibration curves of the 4 compounds and their performance characteristics are presented in Table 6. All of the standards have correlation coefficients of more than 0.99, which showed that all calibration curves were linear over the entire calibration range. Moreover, the LOD and LOQ ranged from 5.13 to 13.78 ng·mL^−1^ and from 13.47 to 41.25 ng·mL^−1^, respectively (Table 6).

The intra-day precision was examined by six repeated injections within a day, and the inter-day variability test was carried out for three continuous days. The relative standard deviation (RSD) values of the intra-day and inter-day variations are from 1.22% to 3.53% and from 0.94% to 3.89%, respectively.

To measure the repeatability, the six varieties of Tartary buckwheat samples were prepared according to the “Extraction procedure” method and were determined for six replicates. In addition, the reproducibility RSD between different tested solutions was all less than 3.03%.

Similarly, the six varieties of Tartary buckwheat samples were tested at 0, 2, 6, 10, 12, and 24 h, respectively, to prove their stability. The RSD values of the stability were all less than 2.98%, indicating that the tested solution was stable within 24 h.

The accuracy of the method was expressed by the recovery. Add the known mixed standard solutions at 3 different concentration levels (80%/100%/120%) into the Chuanqiao No. 1 sample. Recoveries were calculated by the following equation: recovery (%) = 100 × (actual amount–theoretical amount) / spiked amount. The results were evaluated by the relative standard deviation (RSD) values. The recoveries of the four compounds ranged from 95.83% to 106.28%, and RSD ranged from 1.02% to 3.31%.

### 3.6. RNA Extraction and cDNA Preparation

Samples of 50 mg of various varieties of Tartary buckwheat were taken, and total RNA was extracted according to the Plant Total RNA Isolation Kit Plus extraction Kit instruction of Foregene Company. The purity and concentration of RNA were detected with a NanoPhotometer. The extracted total RNA was prepared by reverse transcription system according to the instructions of the RT EasyTM II kit, and the target gene was detected by Real-time PCR EasyTM-SYBR Green I kit. Three key enzyme genes of the flavonoid synthesis pathway, including PAL, 4CL, and ANS, were selected as research objects, and histone coding gene H3 was used as an internal reference gene [23,24]. The primers were synthesized by Sangong Bioengineering (Shanghai) Co., LTD. The primer information is shown in Table 7. The gene expression level was calculated by the 2^−ΔΔCq^ method [25]. 

### 3.7. Analysis of Tartary Buckwheat Seed Coat Color Difference

Seed images were obtained in a microimaging system (Discovry12, Zeiss Company, Germany) and an X-ray digital imaging system (Faxitron MX-20, Faxitro Company, USA) was used to observe the shape of seed embryos and screen high-quality seeds with full seed embryos of buckwheat varieties for subsequent content determination. The seed photos were processed by Affinity Photo processing software, and the seed area was selected to obtain the average RGB ternary color value, which was converted into the grayscale brightness value of human vision. Ternary color values are calculated according to the lightness formula: grayLevel = R × 0.299 + G × 0.587 + B × 0.114 [25].

### 3.8. Data Preprocessing and Statistical Analysis

PLS was used to investigate the meteorological factors on flavonoid content and key enzyme gene expression, and the enzyme gene expression on the seed coat color of Tartary buckwheat. Multiple models are selected to choose from, including the quadratic model, interaction model, and no quadratic model without interaction.

Experimental data were expressed as “x ± s”, the Shapiro–Wilk method was used to judge normality, and Levene’s test method was used to judge homogeneity of variance. In the case of normal distribution, one-way ANOVA was used. If not, the rank sum test was used.

## 4. Conclusions

In the present study, we found that Miqiao No. 1 growing in Liangshan, Sichuan province was the dominant variety of flavonoid content. It can be ascribed to the strongest light intensity in Liangshan (5.14 ± 0.66 KWh/m^2^/day). In addition, light intensity was the key factor affecting flavonoid content and expression of key enzyme genes (light intensity coefficients: 1.17 and 1.51, respectively). The expression levels of PAL and 4CL in the secondary metabolic pathway of flavonoids can promote the accumulation of flavonoids in Tartary buckwheat. Meanwhile, the result showed ANS affects the seed coat color (ANS coefficient: 31.94). The higher the expression of ANS, the darker the seed coat color. On account of PAL, 4CL and ANS share a common substrate “dihydroflavone”; the higher the expression of ANS, the lower the expression of PAL and 4CL, and the higher the anthocyanin content may be. Therefore, we can speculate that the higher the anthocyanin content, the darker the seed coat. These findings provide a theoretical basis and reference for the breeding of buckwheat varieties and their rational utilization of medicinal resources.

## Figures and Tables

**Table 1:** PLS regression model of meteorological factors on flavonoid key enzyme gene expression of Tartary buckwheat.

	1 Comps	2 Comps	3 Comps
X	47.80	63.29	99.8
Y	31.83	39.71	39.8
Regression Equation (1)	Comp_Y_ = 5.383 + 4.447 comp_x_^1^ + 4.184 comp_x_^2^ + 4.179 comp_x_^3^		
CompX Equation (2)	Comp_x_^1^ = 0.22 Tmax − 0.18 Tmin + 0.73 Tdif + 1.51 light intensity + 1.13 Tdif: light intensity		

**Table 2:** PLS regression model of effects of meteorological factors on flavonoid content of Tartary buckwheat.

	1 Comps	2 Comps	3 Comps
X	35.57	60.45	100
Y	46.55	49.28	49.59
Regression Equation (1)	Comp_Y_ = 2.781 + 2.033 comp_x_^1^ + 1.981 comp_x_^2^ + 1.975 comp_x_^3^		
CompX Equation (2)	Comp_x_^1^ = −0.33 Tmax − 0.74 Tmin + 0.76 Tdif + 1.17 light intensity		

**Table 3:** PLS regression model of effects of flavonoid key enzyme gene expression on the seed coat color of Tartary buckwheat.

	1 Comps	2 Comps	3 Comps
X	34.61	89.41	100.00
Y	93.71	95.00	96.06
Regression Equation (1)	Comp_Y_ = 33.88 + 8.503comp_x_^1^ + 7.582 comp_x_^2^ + 6.731 comp_x_^3^		
CompX Equation (2)	Compx^1^ = −4.50 PAL − 3.86 4CL + 31.94 ANS		

**Table 4:** Source information on different varieties of buckwheat.

Varieties	Batch	Sample Number	Producing Area	Harvest Time
Chuanqiao No. 1	20,210,501	S1	Jintang Wufeng town, Chengdu	August 2021
	20,210,502	S2		
	20,210,503	S3		
	20,210,504	S4		
	20,210,505	S5		
Chuanqiao No. 2	20,210,601	S6	Jintang Wufeng town, Chengdu	August 2021
	20,210,602	S7		
	20,210,603	S8		
	20,210,604	S9		
	20,210,605	S10		
Xiqiao No. 1	20,200,601	S11	Jintang Wufeng town, Chengdu	August 2021
	20,200,602	S12		
	20,200,603	S13		
	20,200,604	S14		
	20,200,605	S15		
Xiqiao No. 2	20,200,601	S16	Jintang Wufeng town, Chengdu	August 2021
	20,200,602	S17		
	20,200,603	S18		
	20,200,604	S19		
	20,200,605	S20		
Miqiao No. 1	20,210,701	S21	Liangshan Zhaojue Agricultural Science Institute, Sichuan	July 2021
	20,210,702	S22		
	20,210,703	S23		
	20,210,704	S24		
	20,210,705	S25		
Di ku	20,200,701	S26	Diqing Agricultural Science Institute, Yunnan	August 2021
	20,200,702	S27		
	20,200,703	S28		
	20,200,704	S29		
	20,200,705	S30		

**Table 5:** Parameters of components to be measured by mass spectrometry.

Compound	Ionization Mode	Quantitative Ion Pair (m/z)	Collision Energy (V)
Rutin	Negative	609/271	55.00
Kaempferol-3-O-rutin glycoside	Negative	593/255	52.01
Quercetin	Negative	301/178	16.50
Kaempferol	Negative	285/239	26.31

**Table 6:** The linear calibration curves of four compounds.

Compound	Regression Equation	Correlation Coefficient (r)	Linear Range (μg·mL^−1^)	LOD (ng·mL^−1^)	LOQ (ng·mL^−1^)
Rutin	Y = 66,400X + 541,200	0.9945	0.018~281.20	13.78	41.25
Kaempferol-3-O-rutin	Y = 81,000X + 468,700	0.9923	0.013~202.40	5.13	13.47
Quercetin	Y = 42,900X + 338,900	0.9940	0.018~283.20	8.28	23.89
Kaempferol	Y = 2469.4X + 11,334	0.9930	0.012~201.60	8.98	28.40

**Table 7:** Genes and primers [13].

Gene	Former Primer	Post Primer
**PAL**	ACAAGGCGTTACATGGAGGA	CCAAGCTAGGGTTTCTCCCA
**4CL**	GCAACCATAGACGCTCAAGG	TGCATCGGCTATGGATGGAT
**ANS**	GGAAGTAGGAGGGATAGAGGA	TGGAGAATGAAGGTCAAGGCA

## Data Availability

Not applicable.
